# Analysis of Nipple-Areola Complex Localization Using Male Cadavers: Considerations for Gender-Affirming Surgery

**DOI:** 10.1093/asjof/ojab032

**Published:** 2021-08-25

**Authors:** Amanda K Moorefield, Anna Stock, Zak Rose-Reneau, Pratima K Singh, Zubeen Azari, Barth W Wright, Virender Singhal

**Affiliations:** Anatomy Department Chair, Division of Clinical Anatomy, Kansas City University, Kansas City, MO, USA

## Abstract

**Background:**

Masculinizing chest reconstruction is the most common gender-affirming surgery in transgender males. Despite the current literature’s acknowledgment of the vital role that proper placement of the nipple-areola complex (NAC) plays in a masculine chest contour, there is still much debate regarding the best anatomical landmarks to achieve the desired result.

**Objectives:**

The primary aim of this study is to determine which landmarks for NAC placement can be applied across diverse body types and aid surgeons in creating a masculine chest.

**Methods:**

Twenty-five formaldehyde-embalmed male cadavers were analyzed by conducting various measurements of the NAC, nipple, and surrounding bony and muscular landmarks to identify the most consistent landmarks for proper NAC placement. Linear regression analyses were run to determine how the distance between nipple to respective landmarks varied based on antemortem body mass index (BMI), height, weight, and age.

**Results:**

The measurements for the inferior and lateral borders of the pectoralis major muscle (PMM) displayed the least amount of variance of all the anatomical landmarks studied. Additionally, there was no significant change in these pectoral measurements with varying BMI, height, weight, or age, indicating that these measurements are reliable landmarks for NAC placement across various body types. The average NAC placement in relation to the inferior and lateral borders of PMM was around 2.5 and 2.0 cm, respectively.

**Conclusions:**

Our cadaveric analysis indicates that aesthetically pleasing masculine chest results can be produced consistently across varying body types when adhering to a simple pectoral approach in NAC placement.

There has been a steady increase in the number of gender-affirming surgeries performed in America, with a 12% increase overall from 2019 to 2020.^1^ Masculinizing chest reconstruction, also known as “top surgery,” is the most common surgical intervention performed in transgender males.^2-6^ The Report of the 2015 US Transgender Survey revealed that when transgender men were asked about chest reconstruction, 36% reported having surgery, 61% wanted it someday, 3% were not sure, and 0% said they did not want it.^6^ The chest is the greatest area of discontentment among transgender males, which Skórzewska et al claims is largely attributed to the association between femininity and having breasts.^3-8^ The rate of gender-affirming surgical cases continues to increase as medical associations and academic institutions have begun to recognize gender transition care as a necessity for the psychological well-being of transgender individuals. Chest masculinization specifically has shown to improve social interactions, body image, self-esteem, quality of life, and confidence in one’s sexual identity.^4-12^

Important considerations for a successful surgery include scar placement, final positioning of the nipple-areola complex (NAC), and reshaping of the nipple and NAC.^8^ Masculinization of the NAC has been specifically demonstrated to be a key aspect of chest reconstruction, significantly impacting the physical appearance and psychological well-being of transgender men.^13-26^ There is debate within current literature surrounding which anatomical landmarks provide consistent measurements for nipple and NAC placement while also being applicable across varying body mass indices (BMIs). The primary aim of this study is to determine landmarks for NAC placement that can aid surgeons in creating a masculine chest across diverse body types.

## METHODS

### Study Sample

A study of 25 formaldehyde-embalmed male cadavers was conducted between October 2020 and January 2021. The ages of the cadavers ranged from 57 to 95 (Table 1). Each side of the chest was evaluated separately for a total sample size of 50 NACs. All cadaver specimens were positioned supine for the entirety of the data collection process. This study was conducted in accordance with the guidelines set forth by the Institutional Biosafety Committee (IBC) involving human cadavers. IBC approval was granted by Kansas City University. Additionally, this study adhered to the guidelines set forth by the Belmont Report and informed consent was obtained from all human subjects. Cadavers were utilized on both the Kansas City and Joplin campuses. Inclusion Criteria included males with unaltered chests and with the necessary anatomical landmarks utilized in the study. Exclusion criteria included any surgical alteration of the chest, significant chest deformity, evident gynecomastia, or changes to the NAC region secondary to the embalming process. Physical factors including age, height, weight, and BMI were recorded for the cadaveric sample based on antemortem values. Age, height, and weight were all collected from Kansas City University’s donor charts kept on file. BMI was calculated based on the antemortem height and weight provided. There was one body (2 NACs) that did not have a corresponding specimen height and weight listed in the database. The data were adjusted accordingly to reflect these missing values as presented in Table 1. Of note, though the data were collected using a male cadaver cohort, the figures of the NAC, PMM, and bony landmark measurements were created using live male patients. This was done to show what a surgeon would see in a live patient and further illustrate the clinical and surgical implications of the data.

**Table 1.** Physical Factors of Sample

	N	Range	Mean
Age (yrs)	50	57-95	75
Height (in)	48	65-76	69.6
Weight (Ibs)	48	121-250	170.1
BMI	48	16.6-32.8	24.1

BMI, body mass index.

### Literature Review

Anatomical landmarks were chosen through an extensive literature review. We identified numerous anatomic markers that were mentioned to be reliable predictors for proper NAC placement in chest masculinization surgery. Maas et al, in their literature review, concluded that techniques for NAC localization ranged from a pattern-based technique of chest wall features to equations based on chest and body dimensions.^15^ Landmarks such as the ribs, intercostal spaces (ICSs), or sternal length were recorded as ways to determine the vertical coordinate of the nipple. Landmarks such as the mid-clavicular line, anterior axillary fold, midsternal line, thoracic diameter, or even keeping the nipple at its original horizontal coordinate were listed as well. Additional landmarks such as the inframammary fold, suprasternal notch (SN), manubrium, midaxillary line, tip of xiphoid, Acromioclavicular joint, and umbilicus were also studied. Various distance measurements ranged from simple landmark to landmark distances, ratio of landmarks, or geometric shapes involving the landmarks.^13,15-21,23,25^ Other authors reported using PMM as the main predictor of NAC placement.^13,16-17,19^

### Data Collection

#### Nipple Areola Complex and Internipple Distance

The location of the NAC was first identified using ICS or rib number to identify the vertical coordinate of the nipple. The ICS or rib number that the nipple landed on was recorded. The rib and ICSs were identified through palpation.

**Figure 1.**
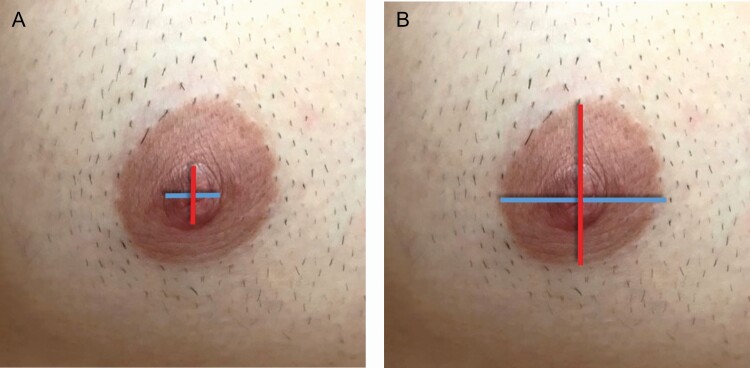
Nipple-areola complex (NAC) and nipple measurements in a 31-year-old male. (A) Red line = nipple height and Blue line = nipple width. (B) Red line = NAC height; Blue line = NAC width.

**Figure 2.**
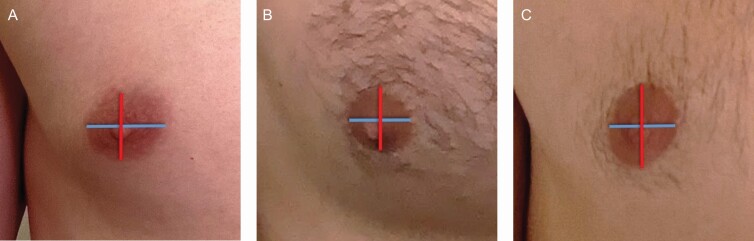
Nipple-areola complex (NAC) shapes from most common on the left to least common on the right. (A) Horizontal oval (width > height) in a 26-year-old male, (B) round (width ≈ height) in a 28-year-old male, and (C) vertical oval (height > width) in a 31-year-old male.

A geometric compass was then used to obtain the nipple and NAC dimensions by placing one end of the compass in the center of the nipple and the other end on the most medial point of the nipple. The compass was then transferred to a metal ruler to record a numerical measurement. These measurements were repeated bilaterally. The same process was repeated to obtain measurements from the center of the nipple to the most inferior portion of the nipple, center of the nipple to the most medial portion of the areola, and center of the nipple to the most inferior portion of the areola (Figure 1A, B). The nipple and NAC measurements were doubled to obtain the ideal height and width, after symmetry was established with a centralized nipple. The shape of the NAC was subsequently recorded as horizontal oval, vertical oval, or round (Figure 2). A horizontal oval shape indicated that the NAC width was greater than height, whereas the vertical oval indicated that height was greater than the width. The criteria for a round NAC were met if the width and height dimension fell within 1 mm of one another.

**Figure 3.**
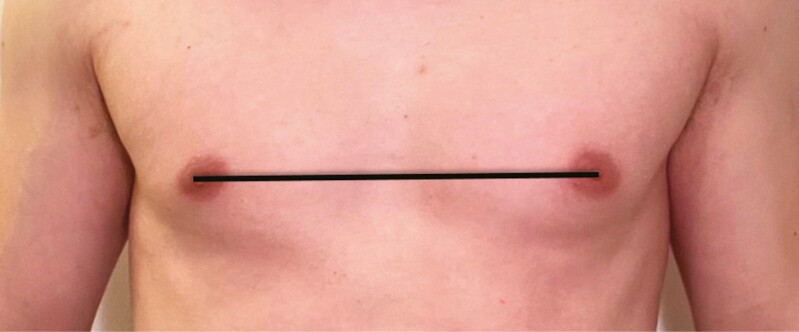
Internipple distance (IND) baseline measurement in a 26-year-old male.

The distance between the center of the 2 nipples was obtained and recorded as the internipple distance (IND) baseline (Figure 3). If certain landmarks were not viable for measurements in a specimen but the NAC was present, then the nipple, NAC, and IND data were still obtained.

#### Bony and Chest Wall Landmarks

**Figure 4.**
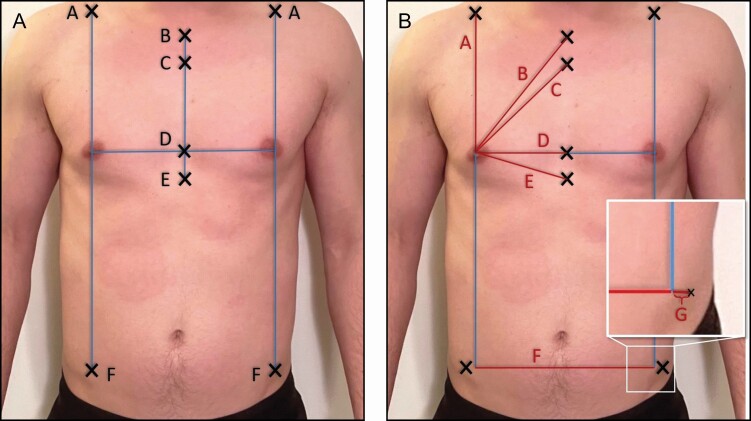
The Xs represent the bony landmarks from a frontal view in a 26-year-old male. (A) A = Clavicular point, B = suprasternal notch, C = Angle of Louis, D = midsternal point, E = xiphoid, and F = ASIS. (B) Interrelated distances between nipple and relevant landmarks: A = nipple-clavicular point, B = nipple-suprasternal notch, C = nipple-Angle of Louis, D = nipple-midsternal point, E = nipple-xiphoid, F = inter-ASIS baseline, and G = ASIS laterality (inset). ASIS, anterior superior iliac spine.

After obtaining the nipple, NAC, and IND baseline data, a series of measurements were obtained for the chest wall and bony landmarks. Identified landmarks included SN, angle of Louis (Louis), xiphoid process, anterior axillary line (AAL), anterior superior iliac spine (ASIS), midsternal point, and clavicular point (Figure 4). The midsternal point is where the IND baseline intersects with the midline of the sternum. The clavicular point is the point directly superior to the nipple drawn at a 90-degree angle from the IND baseline. In the event if the nipple fell lateral to the clavicle, or the clavicle was absent, the measurement was discarded from the sample size. A total of 4 samples were discarded for this reason.

**Figure 5.**
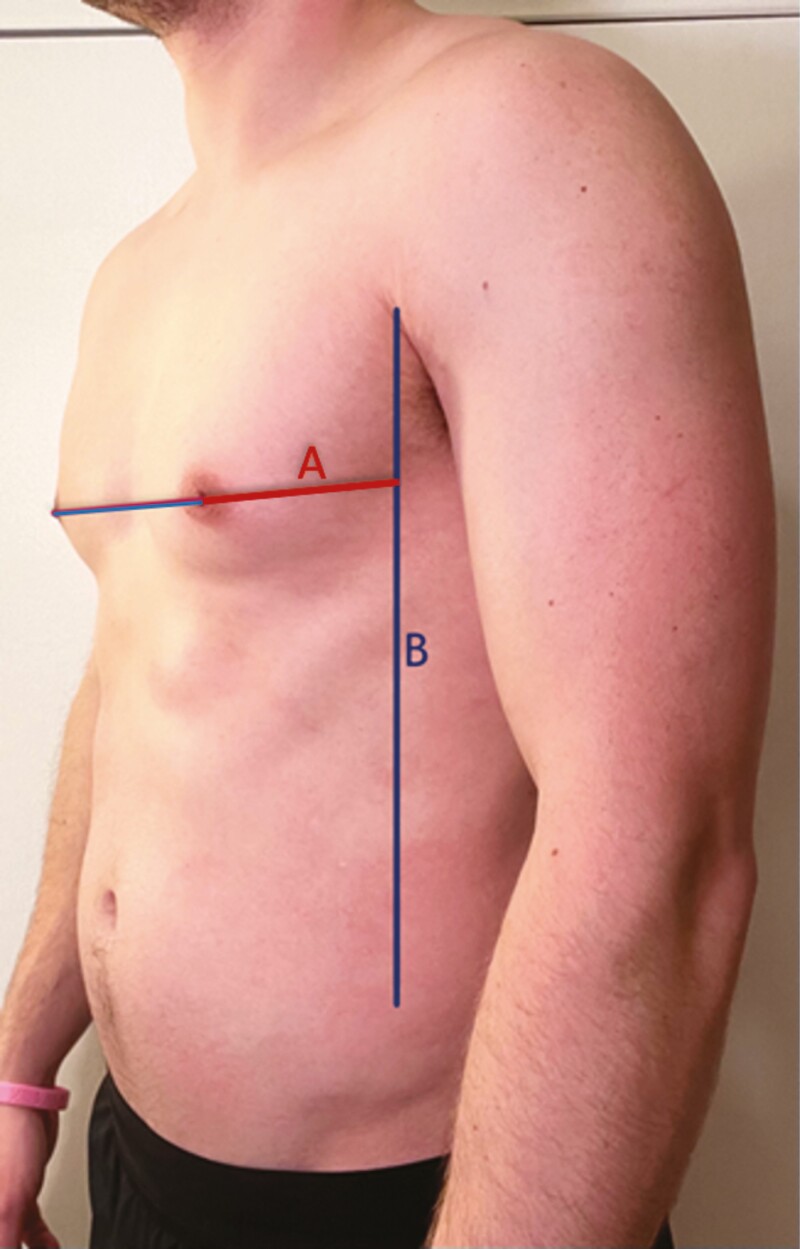
Left oblique view in a 26-year-old male. A = nipple-anterior axillary line and B = anterior axillary line.

Once landmarks were established, measurements were collected. Measurement accuracy and consistency were ensured by placing 6-inch needles in the various regions of interest and then measuring the distance between the needles. This value provided the true distance between 2 points by avoiding measurement variation based on body shape, body positioning, skin folds, or adipose tissue. The following measurements were recorded: nipple-clavicular point, nipple-SN, nipple-Angle of Louis, nipple-midsternal point, nipple-xiphoid, inter-ASIS baseline, and ASIS laterality (as indicated in Figure 4B). Additional measurements that are taken included midsternal point-xiphoid and midsternal point-Louis. Utilizing a surgical marker, the IND was extended bilaterally to mark the AALs (Figure 5).

The ASIS laterality measurement represents the distance that the nipple fell laterally or medially to the ASIS. This measurement utilized a horizontal line connecting the left and right ASIS—the inter-ASIS baseline. A second line was run inferiorly from the center of the nipple at a 90-degree angle from the IND baseline to the inter-ASIS baseline. The distance between the true ASIS and the point along the inter-ASIS baseline corresponding to the vertical axis of the ipsilateral nipple was measured. The majority of the nipples’ vertical axes fell medial to the ASIS. However, in a few instances, the nipples were lateral to the ASIS. In the latter situations, the number was recorded as a negative value. The IND line intersected the sternum superior to the xiphoid process in majority of the bodies; a negative value was used for the nipple-xiphoid measurement if the IND line ran inferior to the xiphoid.

#### *Pectoralis Major* Muscle

Measurements were taken to assess the relationship of the NAC to the PMM. To obtain the PMM measurements, a dissection was first performed to expose the pectoral borders. A superficial incision was made at the SN inferiorly along the midsternal line. Once the costal margin was reached inferiorly, an incision was made laterally toward the AAL. A shallow incision was made from the clavicular notch along the clavicle toward the axilla. The skin and subcutaneous tissue were carefully separated to expose the muscle fibers using blunt dissection. This was done until all borders of the PMM were visible.

**Figure 6.**
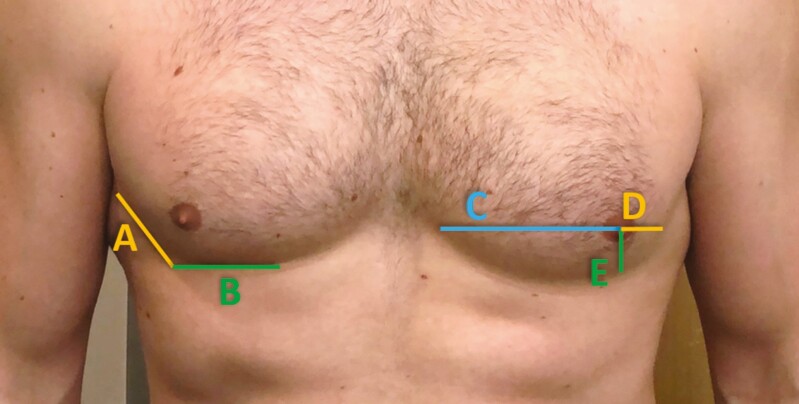
*Pectoralis major* muscle (PMM) measurements in 28-year-old male. A = lateral border of PMM, B = inferior border of PMM, C = nipple-medial border of PMM, D = nipple-lateral border of PMM, and E = nipple-inferior border of PMM.

The dissected skin flap remained connected at the axilla and put back in place after the PMM dissection was completed. With the skin flap in place, a 6-inch needle was inserted through the center of the nipple into the underlying muscle to create a small puncture in the PMM to visualize where the center of the nipple laid on the top of the muscle. The needle was then taken out, the skin reflected, and the needle was placed back into the puncture site. This needle served as a nipple placement marker for the following measurements: nipple to the inferior border of the PMM, nipple to the lateral border of the PMM, and nipple to the medial border of the PMM (where the nipple vertical axis meets the sternum) (Figure 6).

Measurements were gathered using the geometric compass technique that was used previously for the nipple and NAC measurements. Inferior border measurements were taken by measuring the distance from the center of the nipple (the needle) directly inferior to the edge of the PMM. The lateral border was gathered in a similar manner measuring from the needle directly lateral to the edge of the PMM. Finally, the medial border was recorded as the distance from the needle directly medial to the edge of the sternum.

To ensure that the results were valid and reliable, all measurements were taken by 2 researchers. This ensured that potential bias or measurement error was neutralized. The values from researchers 1 and 2 were averaged together for the final recorded value. If there was a difference of more than 0.6 cm noted between the 2 measurements, then each researcher remeasured to avoid potential measurement error.

### Data Analysis

The data were analyzed with IBM SPSS Statistics for Macintosh, Version 27.0 (IBM Corp, Armonk, NY, USA). Descriptive statistics were run on the NAC, nipple, and anatomical landmark measurements to show the maximum, minimum, mean, standard deviation, and variance (Tables 2, 3). Frequency statistics were used to obtain a count of the various NAC shapes as well as the NAC location relative to its associated rib or ICS. Using a classic experimental design model, cause-effect relationship was analyzed using a simple regression analysis for each independent variable. The independent variables or causal agents were the sample demographics: BMI, weight, height, and age. The dependent variables were the anatomic landmark measurements (Table 3).

**Table 2.** Dimensions of Nipple and NAC (Measurements in mm)

Parameters	N	Mean ± SD	Range	Variance
Nipple width	50	8.26 ± 1.83	4.5-12.5	3.36
Nipple height	50	8.13 ± 1.61	5-11.5	2.6
NAC width	50	27.77 ± 5.11	19.5-39	26.15
NAC height	50	23.52 ± 3.83	12.5-32	14.7

NAC, nipple-areola complex; SD, standard deviation.

**Table 3.** Descriptive Statistics of All the Landmarks Listed From Top to Bottom With Least Variance Listed First (Measurements in cm)

Parameters	N	Mean ± SD	Range	Variance
*Pectoralis major*-lateral border	47	2.14 ± 0.68	0.75 to 3.40	0.46
*Pectoralis major*-inferior border	47	2.57 ± 0.99	0.45 to 5.40	0.98
*Pectoralis major*-medial border	47	11.41 ± 1.33	8.65 to 15.45	1.76
Midsternal point	47	11.89 ± 1.34	9.15 to 15.95	1.80
Nipple-angle of Louis	48	14.81 ± 1.40	11.45 to 18.3	1.96
Nipple-suprasternal notch	48	17.46 ± 1.42	14.45 to 21.15	2.00
Nipple-anterior axillary line	48	4.63 ± 1.47	2.40 to 8.15	2.15
Midsternal point-angle of Louis	23	8.31 ± 1.48	4.15 to 10.7	2.19
Nipple-xiphoid	48	14.39 ± 1.67	11.30 to 19.10	2.79
Clavicular point	44	19.15 ± 2.21	14.65 to 23.70	4.87
ASIS laterality	48	2.51 ± 2.38	−2.55 to 10.35	5.67
Midsternal point-xiphoid	24	7.15 ± 2.56	−2.00 to 10.40	6.53
Internipple distance	24	24.41 ± 2.65	20.05 to 30.30	7.04

ASIS, anterior superior iliac spine; SD, standard deviation.

The independent and dependent variables were used to determine if the patient’s physical factors had a significant influence on the anatomic measurements of the sample (Table 4). Before data analysis, assumptions and conditions for regression were verified to be met. Results were deemed significant if the *P*-value was <0.05. Pearson correlations were also used to determine the strength of the relationship between the 2 variables. Correlations between 0.5 and 0.7 were considered to have a fair positive strength of correlation, whereas 0.7 and above were considered to have a strong positive correlation.^27^ For the strong positive values, the correlation coefficient was used to predict the expected change in our dependent variables based on a 1 unit change in the independent variable.

**Table 4.** Results From Linear Regression Statistics Indicating Which Physical Factors Significantly Impacted the Dependent Anatomic Measurements

Parameters	BMI	Weight	Height	Age
Internipple distance	Yes (<0.001)*	Yes (<0.001)*	No	No
NAC height	Yes (0.027)*	No	No	No
NAC width	No	No	No	No
NAC Shape	No	No	No	No
Rib/ICS location	No	No	No	No
Nipple-anterior axillary line	Yes (0.015)*	Yes (0.029)*	No	No
Nipple-suprasternal notch	Yes (<0.001)*	Yes (<0.001)*	No	No
Nipple-angle of Louis	Yes (<0.001)*	Yes (<0.001)*	No	No
Nipple-xiphoid	Yes (<0.001)	Yes (<0.001)	No	No
Clavicular point	Yes (<0.001)*	Yes (<0.001)*	Yes (0.028)*	No
ASIS laterality	No	No	No	No
Nipple to midsternal point	Yes (<0.001)*	Yes (<0.001)*	Yes (0.009)*	No
Midsternal point-xiphoid	No	No	No	No
Midsternal point-angle of Louis	No	No	No	No
*Pectoralis major*-inferior border	No	No	No	No
*Pectoralis major*-lateral border	No	No	No	No
*Pectoralis major*-medial border	Yes (<0.001)*	Yes (<0.001)*	Yes (0.007)*	No

ASIS, anterior superior iliac spine; BMI, body mass index; ICS, intercostal space; NAC, nipple-areola complex;

*Indicates significant *P* values <0.05.

## RESULTS

**Figure 7.**
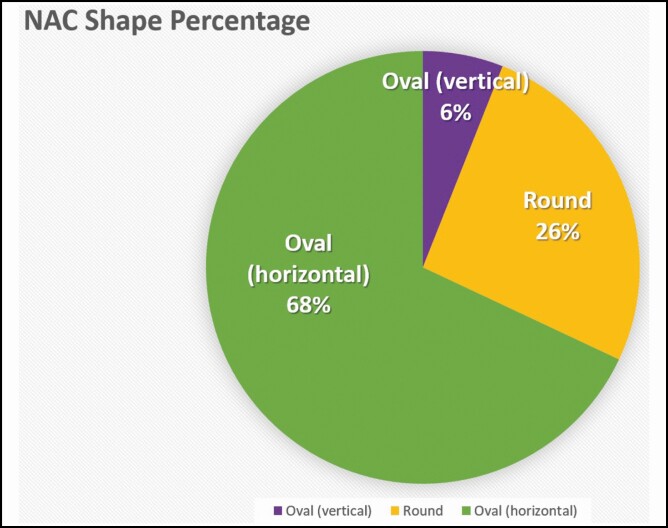
Nipple-areola complex (NAC) shapes by frequency.

**Figure 8.**
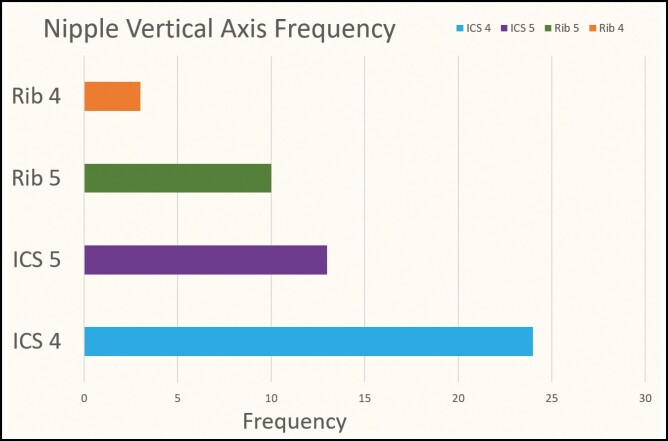
Frequency of nipple-areola complex (NAC) alignment with intercostal space (ICS) or rib level.

The data analysis yielded an average nipple size of 8 × 8 mm (width × height), when rounded to the nearest whole number. Average NAC dimensions were 28 × 24 mm (width × height) (Table 2). Frequency was computed to show the most common NAC shape and with what rib or ICS the NAC fell on or within, respectively. The most common NAC shape was horizontal oval with only a few NACs that were vertical oval. The horizontal oval shape made up 68% (34/50) of the sample with the vertical ovals making up 6% (3/50) for a total oval percentage of 74% (37/50). The remaining 26% (13/50) were round (Figure 7). The most common to least common location of the nipple in relation to ICS/rib was ICS 4, which made up 48% (24/50) of the sample, ICS 5 in 26% (13/50) of the sample, rib 5 in 20% (10/50) of the sample, and rib 4 in 6% (3/50) of the sample (Figure 8). Neither Rib/ICS location nor NAC shape was impacted by the independent factors: BMI, weight, height, or age.

The results were analyzed to determine which landmarks had the lowest and highest variances. A smaller variance was indicative of a more consistent landmark for NAC placement. Out of the bony landmarks tested, the SN and angle of Louis had the smallest variance. Nipple to clavicle, ASIS laterality measurement, and midsternal point to xiphoid measurements had the highest variance.

Body mass index was found to have a statistically significant correlation with IND and NAC height as well as the measurements of the nipple to AAL, SN, angle of Louis, xiphoid, clavicle, and the medial border of the PMM. Weight had a significant correlation with all the same measurements except for the NAC height. BMI had the highest Pearson correlation with the following landmarks in descending order of correlation: IND, nipple to clavicle, nipple to angle of Louis, nipple to midsternal point, medial border of the PMM, nipple-SN, and nipple to xiphoid. All of these values had a positive Pearson correlation of at least 0.55. IND had the strongest positive correlation with BMI at a Pearson correlation of 0.78. Using the linear regression equation and coefficient values, we were able to determine that on average for every 1 unit increase in BMI, the IND increased.44 cm.

Height demonstrated a significant correlation with the nipple to clavicle measurement and the PMM medial border. Pearson correlations were run to analyze the strength of correlation between height and nipple to clavicle measurement, as well as height and the PMM medial border measurement. However, while significant, both results came back with relatively low correlation at .34 and .30, respectively.

The relationship between the lateral and inferior border of the PMM to NAC was not significantly impacted by change in BMI, height, weight, or age. In assessing NAC placement on the chest, the average distance from the center of the nipple to the inferior border of the PMM was ~2.5 cm. The average distance from the center of the nipple to the lateral border of the *pectoralis major* was ~2.0 cm. The average distance from the center of the nipple to the medial border of the sternum was ~12 cm. Additionally, of all the anatomical landmarks studied, the measurements for nipple to inferior and lateral border of the PMM displayed the least amount of variance. The variance for the nipple to inferior border of the PMM was 0.98 cm² (range, 0.45-5.4 cm; SD 0.99 cm), and the variance for the nipple to lateral border of the PMM was 0.46 cm² (range, 0.75-3.4 cm, SD 0.68 cm) (Table 3). The nipple to medial border of the PMM had a higher variance at 1.76 cm² (range, 8.65-15.45 cm, SD 1.33 cm) than the nipple to inferior and lateral borders of PMM.

## DISCUSSION

The main goals of female-to-male chest reconstruction are to remove the breast tissue and excess skin and provide a masculine chest contour with strategically placed incisions to minimize scarring.^5,8^ The NAC placement is a major factor in obtaining a masculine chest contour that is aesthetically pleasing to patients. It is important to not only reshape but also position the NAC to provide a more natural-looking male chest contour. Despite the established importance of proper localization and reshaping of the NAC, there is still much debate in the current literature regarding consistency of landmarks and placement among a diverse patient population. Maas et al did a critical literature review on the ideal NAC placement and identified one of the main flaws in the current body of literature to be the lack of patient diversity, with most of the studies focusing on young, thin patients and some studies identifying obese patients as part of their exclusion criterion.^15,25^ Additionally, they observed discrepancies between studies regarding whether patient physical factors such as BMI, age, weight, and height affected the distances between landmarks.^15^

The findings from our study support the hypothesis that certain physical factors such as BMI, weight, and height impact measurements for the NAC and various anatomical landmarks. Out of these factors, BMI has the strongest correlation with the NAC measurements, indicating change in BMI is strongly linked to changes in the landmark distances. This indicates that BMI is a valuable physical factor to consider when adjusting measurements for NAC placement. Table 5 provides the positive correlations found in our data set ranging from high to moderate correlations and the suggested adjustments that may be considered by surgeons when approaching a patient who falls in an overweight or obese BMI category.

**Table 5.** Pearson Correlation Results for Landmarks With Moderate or Strong Positive Correlation

	Pearson correlation	Significance	Mx*	Y-intercept**	Equation (y = mx + b)	Predicted adjustment
BMI and IND	0.78^a^	<0.001	0.44	13.82	y = (0.44)x + 13.82	Increase .44 cm for every 1 unit increase in BMI
BMI and clavicle	0.69^b^	<0.001	0.32	11.66	y = (0.32)x + 11.66	Increase .32 cm for every 1 unit increase in BMI
BMI and angle of Louis	0.66^b^	<0.001	0.20	9.99	y = (0.20)x + 9.99	Increase .20 cm for every 1 unit increase in BMI
BMI and midsternal point	0.65^b^	<0.001	0.20	7.17	y = (0.20)x+ 7.17	Increase .20 cm for every 1 unit increase in BMI
BMI and PMM medial border	0.63^b^	<0.001	0.19	6.92	y = (0.19)x + 6.92	Increase .19 cm for every 1 unit increase in BMI
BMI and sternal notch	0.57^b^	<0.001	0.17	13.33	y = (0.17)x + 13.33	Increase .17 cm for every 1 unit increase in BMI

BMI, body mass index; IND, internipple distance; PMM, *Pectoralis major* muscle.

^a^Strong correlation,

^b^moderate correlation, x

*independent variable = BMI or height, y

**predicted value for dependent variable.

Age had no impact on any of the measurements. This is important to note, as the average cadaver age in the study was 75 years old, which otherwise could have posed a limitation to the study’s validity. Maas et al concluded that based on their meta-analysis, the most natural shape for a male NAC was horizontal oval, with placement falling somewhere between the fourth and fifth ICS.^15^ Our data further supported this claim. There was no correlation found between age, weight, height, or BMI and the ICS or rib level the NAC was located. We also did not find that any of those factors altered the NAC shape (vertical oval, horizontal oval, or round). Thus, our results support the use of ICS 4 for a horizontal plane and reshaping the NAC to that of a horizontal oval. This also suggests the consistency of these particular landmarks despite the specifics of body positioning, as our analysis was performed on supine bodies.

Ayyala et al presented a simple technique for establishing NAC placement.^18^ They suggested aligning the vertical position of the NAC with the fourth rib and the horizontal position was determined to be one-third of the distance from the AAL to the midline of sternum. Marano et al in their commentary to Ayyala et al’s study questioned a possible limitation to their technique due to patients’ anatomical variation of the chest wall and in body habitus.^18,28^ The measurements of nipple-AAL and nipple-midsternal points that were included in our study allowed us to explore the validity of this technique. Both measurements were shown to vary significantly with changes in BMI and weight. Based on the results of our study, variability in patient physical factors (BMI, weight, height, and age) makes nipple-AAL and nipple-midsternal point measurements less reliable for identifying the proper location of the NAC. However, these could perhaps serve as additional options for ensuring symmetry across the chest wall.

In our series, the medial border of the PMM varied with the independent factors of BMI, weight, and height, yet showed relatively low variance relative to other central landmarks. This medial measurement may be used bilaterally to ensure equal spacing on each side of the chest wall when finalizing NAC placement. The nipple-Louis measurement showed the least amount of variance out of all the bony landmarks, though it also varied with BMI and weight. It also has utility as a main landmark for ensuring equal placement of the NAC bilaterally.

Monstrey et al recommended not adjusting the horizontal plane but raising the vertical plane to be positioned 2-3 cm above the lower border of the PMM.^21^ Agarwal et al determined that the cis-male nipple was on average 2.5 cm medial to the lateral border of the PMM and 2.4 cm above the inferior pectoralis insertion.^16^ Both studies support the reliability of positioning the NAC in relation to pectoral borders and decrease in the range of our estimated distances from the lateral and inferior borders as well.

Maas and Gould questioned how muscular development may impact the NAC to PMM relationship given the difference in anatomy between cisgender males and that of transgender males.^25^ We used the details available in the study of Tanini and Lo Russo to compare our inferior and lateral pectoralis measurements since they did a study to assess the consistency of NAC placement in relation to the PMM.^13,17^ Their data included 2 separate groups of male water polo players both with a BMI range between 18 and 25. Group 1 was the adult men cohort ranging from 23 to 34 years old, whereas group 2 was the teenage cohort ranging from 14 to 16 years old.^17^ We found that our measurements aligned with the teenage cohort. We hypothesized that this could be explained by the inevitable physiologic decrease in muscle tone and mass that occurs with aging, given our older sample size.^29^ Likewise, teenage boys are still going through development and often do not peak in muscle mass until their 20s.^29,30^ Therefore, teenage boys and elderly men, though in different stages of life, are in periods where lean muscle mass is less than that of a healthy, young adult male. This suggests that muscular development does play a role in the relationship between NAC placement and PMM as Maas and Gould suspected.^25^ This is something worth examining further, as patients who have been on testosterone replacement for an extended period before seeking top surgery will have increased muscle mass compared with those who recently began or have never received hormone replacement.

Hormone replacement therapy before top surgery may be beneficial in identifying the projected change in contour of the PMM and allowing for more precise positioning of the NAC. On the contrary, if a patient is seeking top surgery before testosterone replacement, changes in PMM mass, tone, and fat redistribution should be considered as NAC placement may be impacted as predicted from the results of this study. These factors should be included in the preoperative discussion between patients and their physicians to determine the patient’s desired result and specifics for their transition.

Our study supports the consistent relationship between the ideal male NAC and PMM in a population of varying body mass indices (range, 16.6-32.8).^13,17,25^ We assessed for correlation between variable BMI and change in inferior or lateral pectoral measurements but found no significant change across the sample. This indicates that the PMM is a consistent option across varying body types when placing the NAC in its new masculine position. This is important because it indicates a means for NAC localization without having to take into account a patient’s BMI. On the contrary, other techniques must consider BMI adjustment in final NAC placement due to the significant impact a patient’s body type has been shown to have on various anatomical measurements. The need for BMI consideration in general is becoming customary due to the expanding demand for top surgery in various body types and the requisite accommodation for ensuring appropriate NAC placement. Additionally, within the transgender community, potential changes in body habitus must be considered due to fat redistribution secondary to masculinizing hormone therapy. The data collected in this study indicate that the pectoral approach for NAC placement may allow surgeons to bypass any need for BMI adjustment in masculinizing chest surgery and, in doing so, more readily accommodate a broader population.

### Limitations and Future Research

Future research plans include repeating this study on live male patients to evaluate for similar results. Other considerations for this study include investigating trends of pectoral muscular development and its relationship with NAC placement. For example, whether PMM gain during testosterone hormone therapy has a significant impact on lateral, medial, and/or inferior borders across a large sample size. Limitations of this study include that the measurements were performed on cadavers as this could be argued that this changes the body’s natural contour and measurements. Additionally, the premortem physical factors recorded can sometimes be from years before time of death, which could alter assumptions of body type based on the measurements collected in this study. Furthermore, cadavers are not as easily manipulated to obtain measurements in standing or seated positions, so all measurements were taken in supine position. Additional research could also include a similar analysis in human male patients to determine if standing and seated positions yield the same trend.

## CONCLUSIONS

Analysis of the NAC and related musculoskeletal structures in male cadavers indicates that a simple pectoral approach to NAC placement in female-to-male chest reconstruction may be employed to provide an accurate masculine aesthetic. The process involves placing the center of the nipple 2.5 cm above the inferior border of the PMM and 2.0 cm medial to the lateral border of the PMM. The medial border of the PMM and the nipple-midsternal point are both reasonable options to maintain symmetry on each side of the sternum. Additionally, the SN and/or angle of Louis are good bony landmarks for confirming symmetry on each side of the chest wall.

Successful chest masculinization surgery has a profound effect on the physical and emotional well-being of transgender patients, with appropriate surgical NAC placement playing a crucial role in obtaining a masculine chest contour. The data in this study indicate that a reconstructive approach utilizing 2 simple localization steps allows for a successful masculine chest contour in diverse body types.

## Notes

Presented at: 38th Annual Meeting of the American Association of Clinical Anatomists (virtual) in June 2021.
